# Dihydrotanshinone-I modulates Epithelial Mesenchymal Transition (EMT) Thereby Impairing Migration and Clonogenicity of Triple Negative Breast Cancer Cells

**DOI:** 10.31557/APJCP.2021.22.7.2177

**Published:** 2021-07

**Authors:** Akanksha Kashyap, Sheikh Mohammad Umar, Arundhathi Dev J R, Chandra Prakash Prasad

**Affiliations:** *Department of Medical Oncology, Dr. BRA IRCH, AIIMS, New Delhi, India. *

**Keywords:** TNBC, dihydrotanshinone-I, epithelial mesenchymal transition, cell migration

## Abstract

**Background:**

*Salvia miltiorrhiza *Bunge (Danshen), has been used for its therapeutic value in Traditional Chinese Medicine (TCM), for almost a thousand years. Dihydrotanshinone-I (DHTS) is a lipophilic compound isolated from the plant *Salvia miltiorrhiza *that has been shown to induce anti-proliferative and apoptotic effects on breast cancer cells. In the present study, we investigated the anti-migratory effect of DHTS on TNBC cell lines by studying the Epithelial Mesenchymal Transition (EMT) changes.

**Methods:**

IC_50_ values for DHTS in TNBC breast cancer cells were either discovered by literature search or by performing MTT assay. DHTS effect on EMT markers (viz. CD44, E-cadherin, Vimentin, N-cadherin, and active β-catenin) was studied using western blotting. Association between EMT and migration was further carried out in DHTS treated TNBC cells by wound healing assay. Cancer stemness and proliferation potential were further accessed using colony formation assay.

**Results:**

MTT assay revealed IC_50_ of MDA-MB-468 cells at 2 µM for 24 h. Subsequently, DHTS treatment in TNBC cell lines (MDA-MB-468 and MDA-MB-231) led to decrease in mesenchymal markers i.e. vimentin, N-cadherin and, active β-catenin. DHTS treated MDA-MB-468 cells showed a decrease in adhesion protein CD44 and an increase in epithelial protein E-cadherin. Additionally, a decrease in EMT potential was positively associated with the inhibition of migration and clonogenic potential in DHTS treated TNBC cells.

**Conclusion:**

In this study, we have demonstrated for the first time that DHTS has the potential to inhibit the migration and clonogenicity of highly aggressive TNBC cells by obstructing Epithelial to Mesenchymal Transition.

## Introduction

Breast cancer is the most common and leading cause of cancer-associated mortality among women worldwide (Coughlin and Ekwueme., 2009; Bray et al., 2018). Being heterogeneous, breast cancer encompasses distinct morphological features and clinical behaviors. Triple-negative breast cancer (TNBC) is a breast cancer subtype defined by the lack of expression for estrogen receptor (ER), progesterone receptor (PR), and human epidermal growth factor receptor 2 (HER2) (Kittaneh et al., 2013). TNBCs account for 10-20% of all breast cancer globally, but in India, their prevalence is approximately around 25-31% (Sandhu et al., 2013).

TNBCs have been associated with early-onset, higher rates of recurrence, and frequent visceral metastasis (Khaled et al., 2019). The progression of cancer and its subsequent invasion into the surrounding stroma is an essential step towards metastasis, which is the main cause of cancer-associated mortality. Cancer metastasis is largely governed by Epithelial to Mesenchymal Transition (EMT). EMT is a fundamental biological phenomenon wherein polarized epithelial cells that are adherent to the basement membrane lose their polarity by undergoing multiple molecular changes to acquire a mesenchymal phenotype (Kalluri and Weinberg., 2010). This transition enhances the migratory and invasive capabilities of cells by modifying the cell adhesion molecules and ECM components. The cumulating effect results in cancer cells moving out from the primary tumor mass to eventually colonize distant tissue and form metastases (Mittal., 2018). Like other cancer cells, TNBC cells undergo EMT changes for migration and distant dissemination (Al Moustafa., 2013).

In addition to being highly metastatic, TNBC is unresponsive to hormone or receptor-specific therapy due to which surgical resection and cytotoxic chemotherapy remain the primary treatment options. Chemotherapeutic drugs target various areas of tumorigenesis like cell division, apoptosis, angiogenesis, etc (Isakoff., 2010). However, resistance to chemotherapy and unwanted cytotoxicity are major challenges associated with the poor prognosis in TNBCs (Nedeljković and Damjanović., 2019). In this context, the implementation of a complementary treatment might find the solution, as naturally derived compounds offer a great deal of anti-cancer activity. Natural compounds like curcumin, quercetin, berberine, etc have been shown to inhibit TNBC proliferation and aggressiveness (Kundur et al., 2019; El Khalki et al., 2020). In the present manuscript, we investigated the potential of 15,16-dihydrotanshinone-I (DHTS) for its anti-migratory action in TNBC cell lines. (DHTS) is an abietane diterpene compound isolated from the medicinal plant *Salvia Miltiorrhiza*. This bioactive herbal compound has been reported to have several anti-cancer and anti-inflammatory properties (Chen et al., 2019). It is shown to induce apoptosis and autophagy through the mitochondria-mediated caspase-dependent pathway (Wang et al., 2015). Additionally, DHTS has been shown to interferes with Wnt/β-catenin signaling and is involved in Endoplasmic Reticular stress (Chuang et al., 2011). 

In the present study, using two TNBC cell lines i.e. MDA-MB-468 and MDA-MB-231, we have demonstrated that DHTS treatment modulated the EMT markers thereby inhibiting the migration and clonogenicity of TNBC cells, hence suggesting the potential of DHTS in impairing the TNBC cell migration and metastasis.

## Materials and Methods

*Cell Lines*

The MDA-MB-468 and MDA-MB-231 human mammary carcinoma cell lines were procured directly from the NCCS, Pune. All cell lines were grown in DMEM (Gibco, USA) supplemented with 10% FBS, 5 U/ml penicillin, 0.5 U/ml streptomycin, and 2 mM glutamine. 

*Dihydrotanshinone-I (DHTS)*

**Figure 1:**
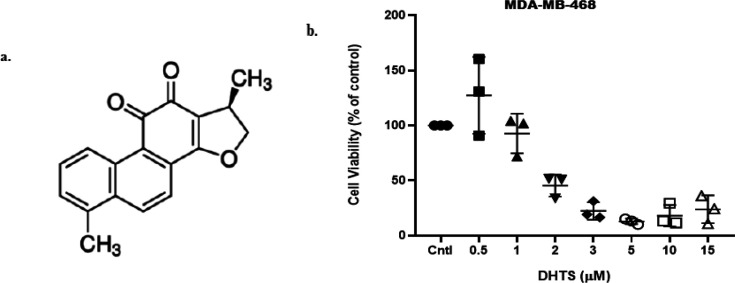
Dihydrotanshinone-I (DHTS) Structure and Cytotoxicity in MDA-MB-468 Cells. (a) Chemical structure of 15,16-dihydrotanshinone I. (b) MDA-MB-468 cells was treated with varying concentrations of DHTS (0-15 µM) for 24 h. Error bars represent the standard error of the mean (n = 3).

DHTS (Cas No (CAS number: 87205-99-0; Empirical Formula: C18H14O3; PubChem Substance ID: 329798655; Mol wt: 278.3) is a red powder extracted from the herb Salvia miltiorrhiza, commonly used in Chinese traditional medicine. For the present study, DHTS [≥98% (HPLC; Cat No. D0947)] was procured from Sigma-Aldrich (MO, USA). The chemical structure of DHTS has been provided in Figure 1a.

*MTT Assay*

MDA-MB-468 cells were harvested by trypsinization and resuspended at a final concentration of 2 × 10^4^ cells/ml in a fresh DMEM medium with 10% FBS. Aliquots of 100 µl cell suspension were plated in 96-well tissue culture plates (Nunc, ThermoFisher Scientific, MA, USA) and cells were treated with a range of DHTS concentrations (0.5–15 µM) using appropriate controls. Cell viability was quantified by the method of Carmichael et al. After 24 h, 10 µl of a 1 mg/ml MTT solution was added to each well, and the plate was incubated for 4 h, allowing viable cells to reduce the yellow MTT to dark-blue formazan crystals, which were dissolved in 100 µl of DMSO. The absorbance in individual wells was determined at 595 nm using a microplate reader (BioTek, VT, USA).

*Western Blotting*

Breast cancer cells i.e. MDA-MB-468 and MDA-MB-231 were treated with DHTS (1 µM and 2 µM) in DMEM supplemented with 1% FBS. Cell were then washed with ice-cold PBS and lysed in ice-cold phosphorylation lysis buffer (PLB;1 M Tris-HCl pH 7.5, 0.5 M NaCl, 30 mM sodium pyrophosphate, 50 mM sodium fluoride, 0.5 M EDTA, 1.5 mM MgCl_2_, 10% Glycerol, and 1% TritonX-100). The protein content of each sample was estimated using the Pierce BCA Protein Assay kit (ThermoFisher, MA, USA). After adjustments for differences in protein content, the samples were suspended in 4X Lammeli buffer, boiled for 5 min, and loaded on 10% SDS-PAGE. After transfer of the separated proteins to PVDF membranes, the membranes were probed with one of these antibodies: CD44 and non-phospho (Active)-β-Catenin (at dilution1:2,000; from Cell Signaling Tech, MA, USA); E-cadherin, Vimentin, and N-cadherin (at dilution1:1,000; from Elabscience, TX, USA); and to β -actin (at 1:10,000; from Sigma, MO, USA) for over-night incubation at 4 °C. After O/N incubation, membranes were extensively washed, and then were incubated with either secondary goat anti-mouse/rabbit HRP-conjugated antibodies purchased from Dako (Glostrup, Denmark). Separated protein bands were visualized using Chemiluminescence HRP substrate (BioRad, CA, USA), and the membranes were imaged and analyzed using the Chemi Doc™ imaging system from Bio-Rad (CA, USA). 

*Wound Healing/Scratch Assay*

TNBC cells were cultured in fresh culture media (DMEM with 10% FBS) to full confluence. The wound was created using a 200 µl micropipette tip and cells were washed with PBS. After that, PBS was replaced with fresh culture media containing DHTS (1 µM) and incubated at 37°C for 48 h. Then, the cells were washed twice with PBS and the wound was observed under a microscope (Leica DM6 Microscope, Wetzlar, Germany). The data were analyzed using ImageJ software (National Institutes of Health, US).

*Clonogenicity Assay*

TNBC cells were treated for 24 hr with 1µM of DHTS. Then, they were collected and 500 cells/well were seeded in 6 well plates. Cells were allowed to grow for one week, till the colonies could be observed. The colonies were fixed with 3.7% formaldehyde for 30 minutes and stained with 0.4% crystal violet. Cells were washed with PBS to remove excess dye. The plate was left to air dry and the colonies were counted using ImageJ software (National Institutes of Health, US).

*3D Culture Assay*

The 3D culture assay was performed as previously published (Lee et al., 2007). Briefly, EHS (Cat # 356234, CORNING, MA, USA) coating was performed on 96 well plates. Single-cell suspension of MDA-MB-231 cells (0.2x106 cells/ml) was mixed with EHS, and 75 µl of this mix was plated over the EHS coating. An appropriate amount of DMEM media was further added and incubated. After visualization of colonies, cells were treated with DHTS (1 µM). Cultures were maintained for 10 days at 37°C in a humidified atmosphere of 5% CO_2_. 

*Statistical Analysis*

SPSS 10.0 for Windows (SPSS Inc., Chicago, IL) was used to perform statistical analysis. All data presented herein are expressed as the mean ± standard error. Each of the experiments was repeated at least three times. Statistical analysis and graphs were generated using GraphPad Prism 5.0 software (CA, USA). Results were considered significant when p values were <0.05.

## Results

*Dihydrotanshinone-I (DHTS) inhibits the proliferation of MDA-MB-468 cells*

Dihydrotanshinone-I (DHTS) has been shown to produce cytotoxic effects on various cancer cell lines, including breast cancer cells (Chen et al., 2019), suggesting that DHTS has the potential to inhibit breast cancer proliferation. In the present study, we evaluated the IC_50_ for MDA-MB-468 cells by MTT assay, where we performed dose-dependent inhibition by DHTS within 24 h. IC_50_ for MDA-MB-468 was found to be at 2 µM (for 24 h, Figure 1b). Moreover, in a previous study, the IC_50_ for MDA-MB-231 was found to be at 1.8 µM (72h) (Hong et al., 2018). Based on previous and present findings, we treated TNBC cells with 1 µM or 2 µM of DHTS depending on the assay performed.

*Dihydrotanshinone-I (DHTS) treatment modulated Epithelial Mesenchymal Transition (EMT) markers in TNBC cells*

**Figure 2:**
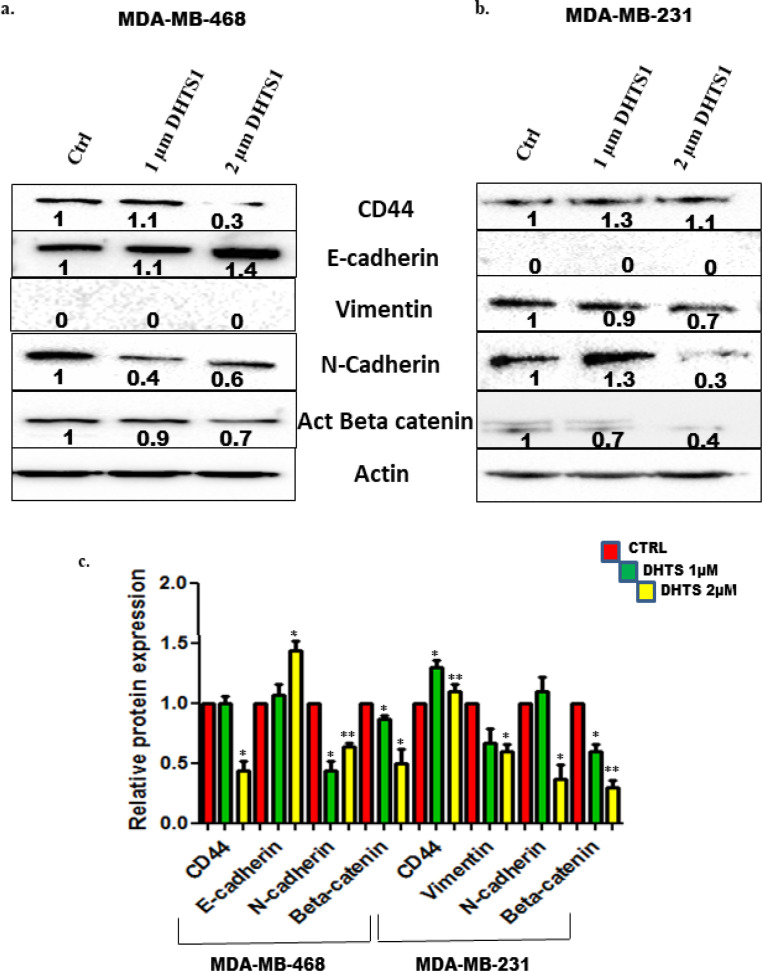
Analysis of EMT Markers in TNBC Cells Treated with DHTS. (a) MDA-MB-468 and (b) MDA-MB-231 cells were treated with DHTS (1 µM and 2 µM) for 24 h and protein expression of CD44, E-cadherin, Vimentin, N-cadherin, and active β-catenin was assessed using western blotting (as described in the Materials and Methods). (c) The quantification of the EMT markers were performed by calculating the integrated densitometric values and normalizing them to the β-actin levels. Statistical comparisons were made with Student’s t-test. All error bars represent the standard error of the mean (n=3). **p = 0.01; *p <0.05

Dihydrotanshinone-I (DHTS) induces its cytotoxic effects on cancer cells by triggering apoptosis (Chen et al., 2019). Additionally, DHTS has been shown to impair the migration of osteosarcoma cells (143B) by downregulating cell adhesion markers like VCAM-1 and ICAM-1 (Chen et al., 2017). In the present study, we hypothesized that DHTS can inhibit /or reverse the EMT status in TNBC cell lines thereby impacting its migration. For experiments, we treated both TNBC cell lines (MDA-MB-468 and MDA-MB-231) with two doses (i.e. 1 µM or 2 µM) of DHTS for 24 h, and expression of EMT markers (viz. CD44, E-cadherin, Vimentin, N-cadherin, and Active β-catenin) were evaluated by western blotting. A marked decrease in the expression of CD44 (adhesion molecule), N-cadherin, and active β-catenin (mesenchymal protein) was observed in DHTS treated MDA-MB-468 cells, when compared with control cells (Figure 2a). On the other hand, a noticeable increase in the E-cadherin (epithelial protein) expression was also observed in DHTS treated MDA-MB-468 cells suggesting that DHTS treatment reverses the EMT status in these cells. Similar findings were also observed in other TNBC cells i.e. MDA-MB-231 when treated with DHTS. There was a significant decrease in the expression of mesenchymal proteins like vimentin, N-cadherin, and active β-catenin, compared with control cells (Figure 2b). Unlike MDA-MB-468 cells, no change in expression of CD44 was observed in DHTS treated cells. Quantification of EMT markers in non-treated and DHTS treated TNBC cells were carried out by calculating Integrated Density Values and normalizing it against β-actin levels (Figure 2c).

Herein, we like to mention that parental MDA-MB-468 cells were negative for vimentin protein, while E-cadherin protein was absent in parental MDA-MB-231 cells, as reported by us previously (Prasad et al., 2016). Overall, these experiments demonstrate that DHTS treatment modulates the EMT markers in TNBC cells that might have potential implications on their migratory behavior.

*Dihydrotanshinone-I (DHTS) treatment significantly impairs the migration of TNBC cells*

**Figure 3:**
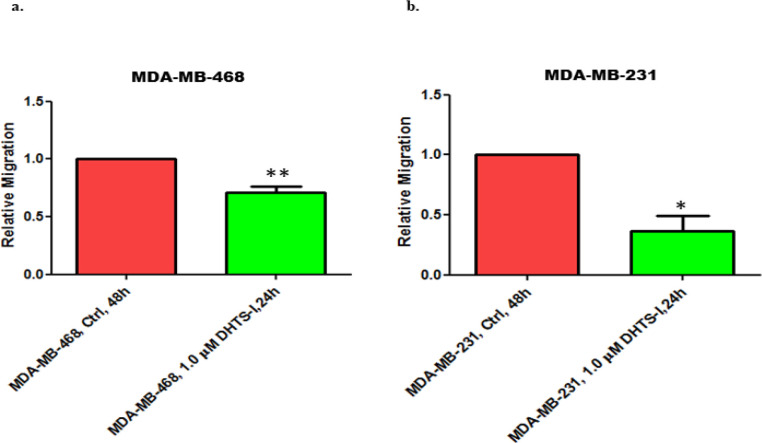
DHTS Treatment Significantly Impaired the Migration of TNBC Cells. (a) MDA-MB-468 and (b) MDA-MB-231 cells were treated with DHTS (1 µM) for 24 h and migration was assessed using Wound healing assay (as described in the Materials and Methods section). Error bars represent the standard error of the mean (n = 3). **p = 0.01; *p <0.05

Epithelial Mesenchymal Transition (EMT) has been associated with cancer cell migration, as the formation of mesenchymal cells and degradation of the basement membrane aids in the migration of cancer cells from the primary site. Hence, EMT positively regulates cell migration. In our previous experiments, we observed that DHTS treatment inhibited the mesenchymal markers in TNBC cells. Additionally, MDA-MB-468 cells showed significant upregulation in the expression of E-cadherin suggesting EMT reversal in these cells. So, we next investigated if DHTS-mediated EMT inhibition of TNBC cells impacts their migration capabilities. Wound healing assay demonstrated significant inhibition of migration of MDA-MB-468 cells (p=0.0029) and MDA-MB-231 cells (p=0.0155) of DHTS in 24 h (Figure 3a and 3b). Overall, these experiments demonstrate that DHTS significantly inhibits the migration of TNBC cells. As inhibition of EMT has been positively associated with stem cell signatures (Kong et al., 2010), we next investigated cancer stemness in TNBC cells after DHTS treatment.

*Inhibition of the clonogenic ability of TNBC cells following DHTS treatment*

**Figure 4:**
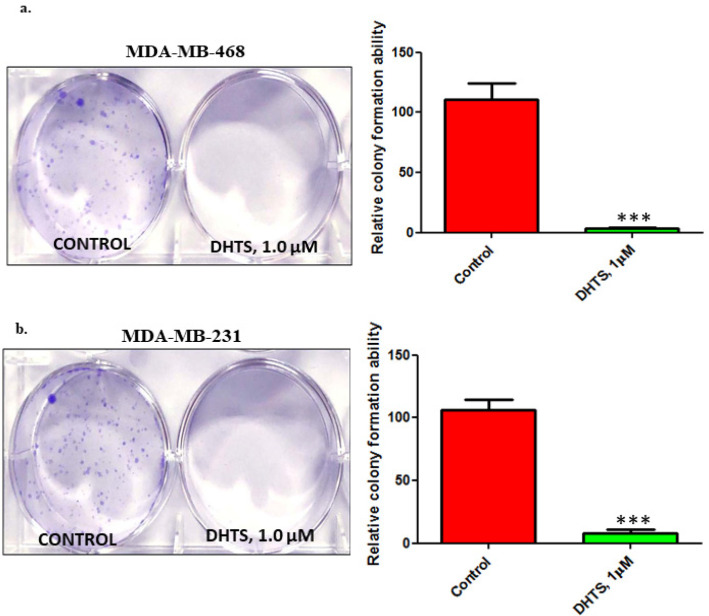
DHTS Treatment Diminishes the Clonogenic Potential of TNBC Cells. Cytotoxic effects of DHTS over a prolonged period in a) MDA-MB-468 and (b) MDA-MB-231 cells by colony formation assay. Both of the TNBC cell lines were allowed to grow for 12 days after treatment with 1 μM DHTS for 24 h. Graphs were generated showing the relative colony formation ability of TNBC cells treated with 1 µM DHTS, compared with controls (Right Panel). All error bars represent the standard error of the mean (n = 3). ***p =0.001

To further examine the cytotoxic effects of DHTS over a prolonged period, clonogenic assays were performed on both of the TNBC cell lines, for 12 days after treatment with 1 μM DHTS for 24 h, respectively. Stained colonies revealed that DHTS treatment for 24 h significantly abrogated the clonogenic ability of MDA-MB-468 and MDA-MB-231 cells respectively (Figure 4a and 4b). The results were consistent with the findings in the cell viability assays and further confirmed the analogous responses of TNBC cells to DHTS treatment.

*DHTS inhibits the growth of MDA-MB-231 in 3D culture*

**Figure 5:**
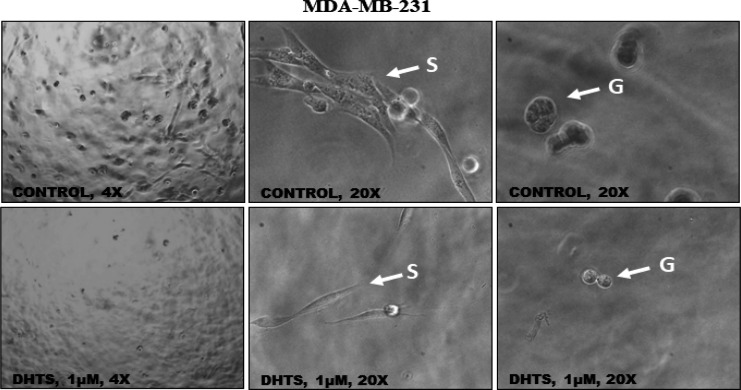
Inhibition of MDA-MB-231 by DHTS in 3D Culture. The upper panel shows bright-field images of the MDA-MB-231 breast cancer 3D model demonstrating stellate (S) and grape-like (G) colonies in untreated (control) cells. The lower panel (DHTS treated) displays a marked reduction in stellate (S) and grape-like (G) colonies (Magnification x20)

MDA-MB-231 cells were grown in 3D culture in presence of 1 μM DHTS. As compared to control cells, DHTS treated wells showed fewer colonies (at low magnification, Figure 5). At higher magnification, we observed MDA-MB-231 as stellate (elongated cell body with the invasive process) and grape-like (characteristic of poor cell-cell adhesion) clusters. DHTS treatment led to a significant reduction in the growth of both stellate as well as grape-like colonies, compared to control cells. The decrease in stellate and grape-like clusters suggests that long-term treatment of DHTS inhibits the invasive potential as well as the proliferation of MDA-MB-231 cells.

**Figure 6:**
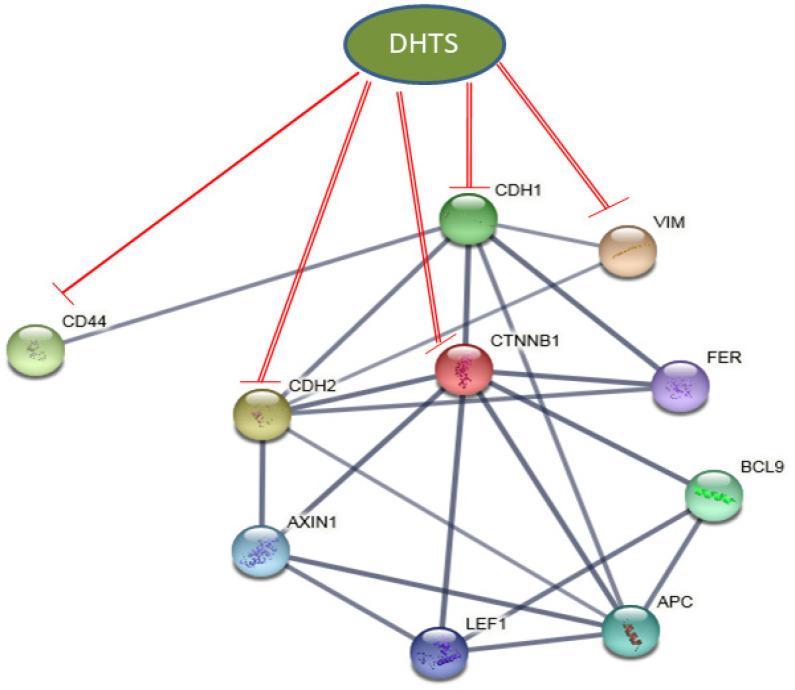
Schematic Diagram Demonstrating DHTS Mediated Modulation of EMT Markers in TNBCs. A protein-protein interaction network was created using the STRING database (involving EMT genes (part of the present study) as well as their close biological interactors. As shown, DHTS might inhibited EMT markers like CDH1 (E-cadherin), CDH2 (N-cadherin), VIM (Vimentin) by modulating the expression on CTNNB1 (β-catenin) in TNBC cells. In the interactome, the CD44 gene was found to be interacting with only CDH1 (E-cadherin), and that might be the reason why DHTS inhibited CD44 protein in MDA-MB-468 cells (positive for E-cadherin) and not in MDA-MB-231 cells (negative for E-cadherin). The other interacting genes like AXIN1, LEF1, BCL9, and FER must be investigated for their role in the EMT process in TNBCs

## Discussion

Breast cancer is the most common malignancy in women in developing as well as developed countries (Ghoncheh et al., 2016). Approximately, one-third of breast cancers are triple negative breast cancer (TNBC), which are often associated with a poor prognosis. TNBCs are highly migratory and invasive, as around 30% of patients have a higher risk of metastasis within three years of initial diagnosis. Hence, it is important to identify new drugs that can target tumor cell migration and metastasis. In the present study, we worked with Dihydrotanshinone-I (DHTS), a lipophilic compound isolated from the plant Salvia miltiorrhiza. *Salvia miltiorrhiza *Bunge (Danshen), has been used for its therapeutic value in Traditional Chinese Medicine (TCM), for almost a thousand years. It is used to treat hepatitis, Inflammation, cardiovascular disease, and cancer (Tsai et al., 2007; Wang et al., 2007). 

Using two breast cancer cell lines (i.e. MCF-7 and MDA-MB-231), Tsai et al., (2007) demonstrated that DHTS significantly inhibits the proliferation of breast cancer cells by modulating the cell cycle genes i.e. cyclin D1, cyclin D3, cyclin E, and CDK4. Additionally, they found that DHTS induced apoptosis in these cells through the mitochondrial pathway (Tsai et al., 2007). In other study, DHTS has been shown to inhibit cancer stem cells (CSCs) in TNBC cell line i.e. MDA-MB-231 by activation of NOX5 mediated ROS/Stat3/IL-6 pathway (Kim et al., 2019). Recently, DHTS has been shown to inhibit the migration of ovarian cancer cells through the modulation of PI3K/AKT pathway (Wang et al., 2020). On these lines in the present study, we also hypothesize that DHTS treatment might inhibit the migratory behavior of TNBC cells by impeding EMT, as to date, there are no reports of DHTS on TNBC cell migration in association with EMT changes. Our present findings showed that DHTS inhibited the EMT process in both the TNBC cell lines (i.e. MDA-MB-468 and MDA-MB-231). A significant reduction in the expression of mesenchymal proteins i.e. N-cadherin and active β-catenin was observed in both the TNBC cell lines treated with DHTS. Furthermore, DHTS treated MDA-MB-468 cells showed a reduction in CD44 protein, as well as a marked increase was observed in the expression of epithelial protein E-cadherin. However, no such changes were observed in DHTS treated MDA-MB-231 cells. Based on our present results, we have provided a schematic diagram of how DHTS modulates the EMT markers in TNBC cells (Figure 6). As, both TNBC cell lines responded to DHTS treatment and modulated substantial EMT markers, we next investigated the effect of DHTS on TNBC cell migration. 

Our wound healing experiment showed that DHTS treatment (1µM for 24 h) significantly inhibited the TNBC cell migration thereby validating our EMT findings. Inhibition of EMT and migration by DHTS put forward the notion that DHTS like other plant-derived polyphenols i.e. curcumin and quercetin harbor anti-migratory action. As DHTS has been shown to inhibit the proliferation of breast cancer cells previously, in the present study we evaluated the cytotoxic effects of DHTS over a prolonged period by performing clonogenic assays on both of the TNBC cell lines. Our results demonstrated that DHTS treatment for 24 h thoroughly abrogated the clonogenic ability of MDA-MB-468 and MDA-MB-231 cells suggesting DHTS likely the stemness and proliferative potential of TNBC cells. These findings were further validated by us in 3D culture experiments (mimicking the physiological context), where we demonstrated that DHTS treatment markedly reduced the proliferation and invasion of MDA-MB-231 cells. 

In the present study, we have demonstrated for the first time that Dihydrotanshinone-I (DHTS) inhibits the migration of highly aggressive TNBC cells by obstructing Epithelial to Mesenchymal Transition (EMT). Additionally, DHTS treatment impaired the clonogenic potential of TNBC cells thereby advocating DHTS in the effective treatment of TNBC alone or in combination with existing chemotherapeutic drugs.

## Author Contribution Statement

CPP and AK designed the study. AK, SMU, and ADJR are involved in experiments and the acquisition of data. AK and CPP analysed the data. AK, SMU, ADJR, and CPP drafted the manuscript. All authors have read and approved the final version of this manuscript.
